# Memphis Medical School

**Published:** 1846-03

**Authors:** 


MEMPHIS MEDICAL SCHOOL.

The papers of Tennessee announced, some time ago, that a charter had been obtained for a Medical School in Memphis. A few days since we saw a letter from a physician of that city, from which it appears that arrangements are in progress for putting the School into early operation. He spoke of having obtained a charter, but did not mention who were to be his associates in the enterprise. Memphis promises to become a great city at some future day, and doubtless will be the seat of a flourishing school of medicine. The immediate success of the projected institution will depend upon the character and extent of its equipments.

---

VACCINE APHORISMS.

Vaccination, at the present time, is attracting an unusual share of public attention, from the circumstance that small pox has obtained a very uncommon prevalence. We adverted to this fact in our last number. Since that time several cases of the disease, but no fatal ones, we believe, have appeared in this city, and we learn that it continues to prevail, in a modified form, in the Atlantic cities. The following aphorisms, copied by the Boston Medical and Surgical Journal from a New Hampshire paper, are sanctioned by medical men the most experienced in vaccination. We have no doubt they will be acceptable to our readers:

"1. A 'scar' of the cowpox, having all the characteristic marks, and perfectly distinct, is not to be relied upon as an evidence of certain protection, unless proved by re-vaccination.

"2. *Complete* vaccination forever secures an immunity to the individual from infection of the smallpox, varioloid, and from specific effects of cowpox.

"3. In primary successful vaccinations, there is little evidence of the operation of the virus before the fifth or sixth day, while in subsequent vaccinations, if the first be *complete*, there is speedy inflammation and itching, and the whole disappears at about the time that the first should distinctly appear.

"4. The most rigid scrutiny is requisite in the selection of matter, that it be collected at the right period of the vesicle, and from individuals of robust health, free from any cutaneous or other disorders, and of these co ditions the physician is the only competent judge.

"5. Cutaneous diseases (as perhaps visceral and other disorders,) seriously modify the character of the genuine vaccine affection, both as it regards the pur ty of the virus, for transferring the disease, and also the degree of security afforded the vaccinated, in consequence of impaired susceptibility.

"6. *Re-vaccinate in all cases*, and repeat the operation so long as it specifically affects the system."

---

### MEDICAL CLASSES.

From the published catalogues, we learn that there were last winter in the Willoughby Medical School 164 students, in the Medical Department of Transylvania University 170, in the Ohio Medical College 192, in the Medical Institute of Louisville 345, in Jefferson Medical College 469, and in the University of Pennsylvania 471. We hope the deans of faculties of the several other schools will forward to us catalogues so soon as they are published.

---

